# Driving the implementation of hospital examination reservation system through hospital management

**DOI:** 10.1186/s12913-023-10467-x

**Published:** 2024-01-09

**Authors:** Qi Wang, Yingjie Ma, Jian Mao, Jingyan Song, Mingzhao Xiao, Qinghua Zhao, Fang Yuan, Lei Hu

**Affiliations:** 1 Information Center, The First Affiliated Hospital of Chongqing Medical University, Chongqing, China; 2 Department of Medical Research, Daping Hospital, Third Military Medical University, Chongqing, China; 3 Medical Department, Yidu Cloud (Beijing) Technology Co., Ltd, Beijing, China; 4 Department of Nursing, The First Affiliated Hospital of Chongqing Medical University, Chongqing, China; 5 Department of Urology, The First Affiliated Hospital of Chongqing Medical University, Chongqing, China; 6 Department of Epidemiology and Health Statistics, School of Public Health and Management, Chongqing Medical University, Chongqing, China

**Keywords:** UTAUT2, Hospital examination reservation system, Hospital management, Behavioral intention, Habit, Innovation

## Abstract

**Background:**

Hospital Examination Reservation System (HERS) was designed for reducing appointment examination waiting time and enhancing patients’ medical satisfaction in China, but implementing HERS would encounter many difficulties. This study would investigate the factors that influence patients’ utilization of HERS through UTAUT2, and provide valuable insights for hospital managements to drive the effective implementation of HERS. It is helpful for improving patients’ medical satisfaction.

**Methods:**

We conducted a survey through the Sojump platform, targeting patients were who have already used HERS. We collected questionnaire information related to factors behavior intention, performance expectancy, and effort expectancy. Subsequently, we employed a structural equation model to analyze the factors influencing patients’ utilization of HERS.

**Results:**

A total of 394 valid questionnaires were collected. Habit was the main direct positive factor influencing the behavioral intention of HERS (β = 0.593; 95%CI: 0.072, 1.944; *P* = 0.002), followed by patient innovation (β = 0.269; 95%CI: 0.002, 0.443; *P* < 0.001), effort expectancy (β = 0.239; 95%CI: -0.022, 0.478; *P* = 0.048). Patient innovation and facilitating conditions also have an indirect effect on behavioral intention. Perceived privacy exposure has a significantly negative effect on behavioral intention (β=-0.138; 95%CI: -0.225, -0.047; *P* < 0.001). The above variables explained 56.7% of the variation in behavioral intention.

**Conclusions:**

When HERS is implemented in hospitals, managements should arrange volunteers to guide patients to bring up the habit and solve the using difficulties, and managements could invite patients with high innovation to recommend HERS to others, what’s more, it is a valid way to retain the old form of appointment to pass the transition period to the new system. HERS utilization and patients’ medical satisfaction will be enhanced through the guidance of hospital management means.

**Supplementary Information:**

The online version contains supplementary material available at 10.1186/s12913-023-10467-x.

## Background

In recent years, China’s social informatization had shown a trend of rapid development, and safer, more effective and more intelligent Hospital Information System(HIS) had become the key targets of the construction of smart hospitals, which aimed to improve patient satisfaction and provide high-quality services [1]. There was growing evidence that increased patient safety, improved clinical effectiveness, and higher hospital reputation were associated with higher patient satisfaction [2]. Long waiting times of appointment examination had been identified as a major cause of low patient satisfaction [3–5]. Due to the backward informatization in developing countries and the large number of patients, it often took a longer time for appointment examination in China [6]. In a systematic review, 60% of the studies found that long waiting times was the most unsatisfactory factor for Chinese patients [7]. Therefore, how to reduce patients’ waiting time became the key to improve medical satisfaction, almost all of the medical institutions were trying to find an effective method to control the service process for patients, such as reservation strategy and scheduling rules.

Hospital Examination Reservation System (HERS) based on HIS was considered an effective method at present. Nowadays, HERS has been widely used in Chinese 3 A hospitals, and it is helpful for outpatients to arrange examination time. Although patients could book examination both through HERS and doctors, the system is advocated because doctors were always busy. HERS recommended the most effective examination sequence and time to patients through the data mining technique, and it could be used to check in, modify the appointment examination time and print the examination report. Relevant studies had shown that HERS will significantly reduce the waiting time [1, 8], and it is effective to improve patients’ medical satisfaction.

However, the new system will be judged a success only if it was widely used [9]. The implementation of HERS had encountered many difficulties, patients were used to getting help from their doctors to reserve examination and it was troublesome for them to learn to use a new system [10]. Ahlan and Ahmad thought that more than half of the information systems were not being used in developing countries because additional time required to enter patient records and review decisions provided by the system, and user and staff resistance [11]. Handayani et al. also thought that doctors and nurses do not make good use of the HIS [12]. At present, most studies focused on the advantages and benefits of a new system, but there were few studies focusing on the use intention of the system in developing countries [11]. The successful implementation of HERS could not only optimize the hospital examination process, but also improve the overall utilization rate of hospital examination equipment [13], and it would greatly improve patients’ medical satisfaction. So it is important for hospitals to improve the utilization of HERS.

Related studies suggested that support from management was critical to the sustainability of HIS implementation [12], managements’ attitudes about acceptance or rejection of the HERS would influence user’s attitudes. and they could lay down regulations to promote the implementation. Nowadays, there are no studies focus on promoting the implementation of HERS, so it is not clear that how to increase the utilization of HERS. This work will explore the factors that influence patients’ use of HERS through the Unified Theory of Acceptance and Use of Technology 2 (UTAUT2), and provide references for hospital managements to promote the HERS implementation in hospitals. And this will help to improve the ability of hospital management and patient’s medical satisfaction.

## Methods

### Theory and hypothesis

**Fig. 1:**
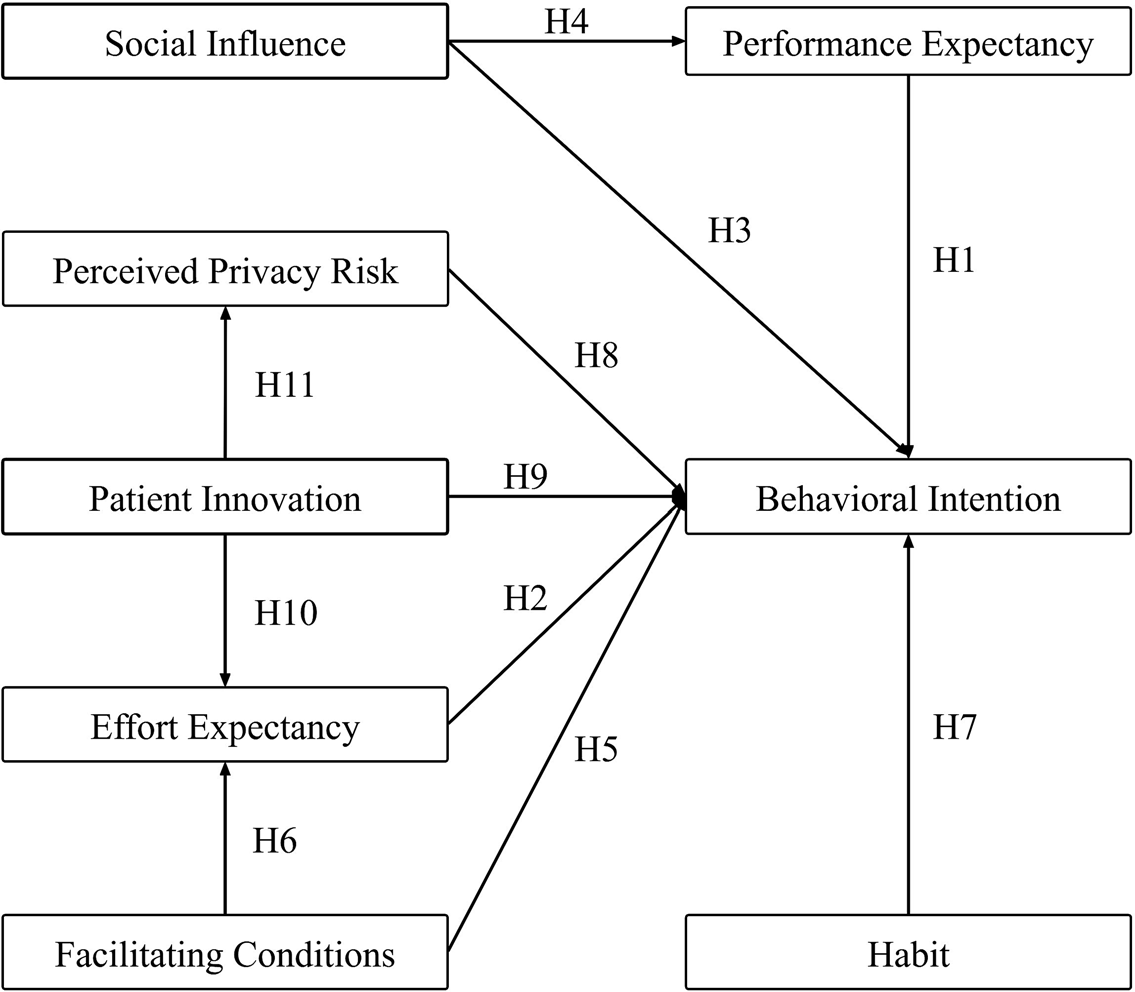
The proposed model

Venkatesh et al. proposed the UTAUT2 in 2012. According to the UTAUT2, performance expectancy (PE), effort expectancy (EE), social influence (SI), facilitating conditions (FC), hedonic motivation, price value, and habit (HT) was the factors of behavioral intention (BI), and the model could explain 70% of the variation in BI [14]. Nowadays, UTAUT2 had been widely used to explain the reason of why a new system is well implemented, which significantly helped improve practical use, and many researchers had extended the model in their research. Hoque et al. added technology anxiety and resistance to change to study the factors affecting the intention of the elderly to adopt mobile health(mHealth) services [15]. Wang et al. believed that task-technology fit will positively affect the intention to use healthcare wearable devices [16]. Prasetyo et al. found learning value and instructor characteristics can affect the acceptance of medical education elearning platforms during the COVID-19 pandemic [17]. According to relevant literatures, the following hypotheses are proposed in this study, and the proposed model is shown in Fig. 1.

PE refers to the benefit patients expect from HERS, such as shortening waiting time and improving examination efficiency. The greater the benefit is, the higher behavioral intention patients will have, so hypothesis 1 is established:

#### H1

PE has a positive effect on BI of HERS.

EE refers to the degree of the ease for patients to learn to use or use the HERS. The easier the using is, the higher BI patients will have, thus establish hypothesis 2:

#### H2

EE has a positive effect on BI of HERS.

SI refers to the degree of support from doctors and patients’ relatives and friends for using HERS. The higher the degree of support is, the higher BI patients will have, so hypothesis 3 is established:

#### H3

SI has a positive effect on BI of HERS.

In a study of web-based interactive self-management techniques, SI was found to indirectly affect BI through PE [18], and the products recommended by doctors will be highly expected by patients [19]. The higher the degree of support is, the higher PE patients will have, so hypothesis 4 is established:

#### H4

SI has a positive effect on PE of HERS.

FC refer to the adequacy of the necessary resources for using HERS, such as mobile phones and Internet. The more sufficient the resources are, the higher BI patients will have. Therefore, hypothesis 5 is established:

#### H5

FC has a positive effect on BI of HERS.

Studies have shown that when users find it convenient to use a system (they can get help from others quickly), the perceived difficulty is reduced [16, 20]. The more convenient using HERS is, the easier patients perceive using system will be. Therefore, hypothesis 6 is established:

#### H6

FC has a positive effect on EE of HERS.

HT refers to the habitual or automatic behavior of patients using the HERS, which presents the results of previous experience. Once an action becomes a habit, it is automatic and does not require a conscious decision [14]. HT is always considered as a predictor of actual use because its influence on actual use exceeds BI, our study does not collect actual use data, so hypothesis 7 is established:

#### H7

HT has a positive effect on BI of HERS.

HERS can improve patients’ satisfaction with medical treatment, but such information system will collect patients’ demographic information and case data, and there is a risk of data leakage. Multiple studies have shown that perceived privacy risks (PPR) affect people’s use of system [19, 21, 22]. The higher the degree of perceived risk is, the lower BI patients will have, so hypothesis 8 is established:

#### H8

PPR has a negative effect on BI of HERS.

Patients innovation (PI) is the personal characteristic of patients. Rogers called the process of innovation adoption from the first contact with information about innovative products to the final adoption of new products. Innovators are bold, adventurous and keen on new ideas and concepts [23]. The more innovative patients are, the higher BI they will have. Therefore, hypothesis 9 is established:

#### H9

PI has a positive effect on BI of HERS.

Self-efficacy is the confidence that people will take the necessary actions in order to achieve their desired goals [24]. Research shows that many innovative ideas and behaviors are based on people’s confidence, and self-efficacy is an important factor affecting PI [23, 25–27]. The more innovative patients are, the more confident they will be in using the system, which means patients perceived that it was easier for them to use the HERS, so hypothesis 10 is established:

#### H10

PI has a positive effect on EE of HERS.

Midgley et al. believed that innovators were more willing to take the risk of using a new system [28]. According to the Diffusion of Innovations, innovators are bold and adventurous [23], therefore, under the same degree of risk, patients with strong innovation have a lower degree of PPR, so hypothesis 11 is established:

#### H11

PI has a negative effect on PPR of HERS.

### Data collection

The target population of this study was patients who had used the HERS. Six months after implementing the system, the questionnaire investigator was commissioned to collected the behavioral intention data after patients finished using the HERS with the way of simple random sampling, and the questionnaire could be seen from Table 1. Questionnaire was filled through the Sojump (Changsha ran Xing InfoTech Ltd, China). Sojump is a professional online questionnaire survey tool focusing on providing users with powerful and humanized online questionnaire design, data collection, custom reports, survey results analysis and other services. Compared with traditional survey methods and other survey websites or survey systems, Sojump has obvious advantages of fast, easy to use and low cost, and has been widely used by a large number of enterprises and individuals in China. The items were measured with a 5-point Likert scale ranging from “strongly disagree” (1) to “strongly agree” (5). Before the survey, we introduced the purpose of the study and filled in the questionnaire with the consent of the patients. The questionnaire was filled out by the patients themselves, and each WeChat account and mobile IP address could complete the questionnaire only once.

**Table 1:** Questionnaire

Construct	Item	Question
PE^a^	PE1	I found HERS very helpful.
	PE2	HERS helped me to complete the appointment examination faster.
	PE3	HERS had improved my appointment examination efficiency.
EE^b^	EE1	I found learning to use the HERS very easy.
	EE2	I found using HERS very easy.
	EE3	The instructions on the HERS were clear and they are easy to understand.
SI^c^	SI1	My family suggested me to use the HERS when booking examination (when I need to change my appointment).
	SI2	My friends and colleagues suggested me to use the HERS when booking examination (when I need to change my appointment).
	SI3	The doctor and nurse suggested me to use the HERS when booking examination (when I need to change my appointment).
FC^d^	FC1	HERS was widespread used.
	FC2	I had the necessary tools to use the HERS (such as mobile phone, WeChat, Internet, hospital examination reservation machine, etc.)
	FC3	I had the necessary knowledge to use the HERS and I could use it.
HT^e^	HT1	HERS was similar to some of the systems I had used.
	HT2	I could get help from others when meeting troubles in using HERS.
	HT3	It had become the habit for me to use HERS.
PPR^f^	PPR1	If I use the HERS, my personal privacy will be disclosed.
	PPR2	If I use the HERS, my information will be used for other purposes.
	PPR3	My personal privacy would be stolen and abused by cybercriminals.
PI^g^	PI1	If I find the new technology, I will try it (e.g., HERS).
	PI2	I’ll be the first in my friends to try the new technology.
	PI3	I’m eager to try new technologies (e.g., HERS).
BI^h^	BI1	I plan to continue using HERS in the future.
	BI2	I will always use HERS when booking examination.
	BI3	I would recommend other patients to use HERS.
	BI4	In general, I would like to use the HERS.

^a^PE: performance expectancy, ^b^EE: effort expectancy, ^c^SI: social influence, ^d^FC: facilitating conditions, ^e^HT: habit, ^f^PPR: perceived privacy risks, ^g^PI: Patients innovation, ^h^BI: behavioral intention

### Data analysis

The demographic characteristics of patients were analyzed by using the SPSS (Version 24.0. Armonk, NY: IBM Corp; 2021). The continuous variables were expressed by mean and standard deviation, and the discrete variables were expressed by number of cases and percentage. AMOS26.0 (Version 26.0. Armonk, NY: IBM Corp; 2021) was used to evaluate the structural equation model (SEM) which include the measurement model and the structural model. The measurement model is mainly used to analyze the representation of items to constructs and the relationship between constructs. Reliability was measured by Cronbach’s alpha and composite reliability (CR), both of which needed to be greater than 0.70. We measured the validity of convergence based on the average variance extracted (AVE), and a value higher than 0.50 indicated that the construct had good convergence. The discriminant validity was acceptable if the correlation coefficient between structures was less than the square-root of the corresponding structure.

The structural model is mainly used to test pre-established assumptions. For the structural model, 2,000 bias-corrected samples were extracted to calculate the path coefficients and their significance. P-value (two-tailed) less than or equal to 0.05 was considered to have significant effects. R^2^ was used to represent the explanatory degree of the independent variables with respect to the dependent variables. The model fit was generally considered acceptable if χ^2^/df was less than three; the goodness of fit index (GFI), normed fit index (NFI), comparative fit index (CFI) and the incremental fit index (IFI) were all above 0.90, and the root mean-squared error of approximation (RMSEA) was less than 0.08.

Compared with linear regression model, SEM can be used to verify the pre-established theoretical model and reflect the causal relationship between variables, what’s more, indirect effects between variables can also be measured. The sample size required for SEM is 5 times the number of free parameters and 10 times the number of observed variables, so a sample size of at least 250 is required for this study.

## Results

### Demographic characteristic

A total of 394 valid questionnaires were collected in this study, among which 279 (70.81%) patients were female, 251 (63.71%) patients had a high school education level or above, 283 (71.83%) patients were urban residents, and the average age of patients was 44.05 ± 16.66 years. 84 patients (21.32%) found it is difficult to use HERS. The overall Cronbach’s alpha of the questionnaire is 0.956.

### The measurement model

Two indexes with low factor loading (HT4 and PI4) were removed from the model. It can be seen from Table 2 that AVE, CR and Cronbach’s alpha are all greater than recommended values, which indicates good reliability and convergent validity. The discriminant validity of each construct is shown in Table 3. Although correlation coefficients of 3 constructs (SI vs. FC, SI vs. HT and FC vs. HT) are greater than the square root of corresponding AVE, the other correlation coefficients meet the requirements, so discriminant validity is still considered acceptable, and the data collected could establish the structural model well.

**Table 2:** Convergent validity and reliability analysis

Construct	Item	Factor loading	AVE^a^ (> 0.5)	CR^b^(> 0.7)	Cronbach’s α (> 0.7)
PE^c^	PE1	0.945	0.891	0.961	0.960
	PE2	0.958			
	PE3	0.929			
EE^d^	EE1	0.894	0.840	0.940	0.940
	EE2	0.929			
	EE3	0.926			
SI^e^	SI1	0.820	0.717	0.910	0.922
	SI2	0.829			
	SI3	0.863			
	SI4	0.873			
FC^f^	FC1	0.833	0.698	0.902	0.891
	FC2	0.909			
	FC3	0.808			
	FC4	0.787			
HT^g^	HT1	0.907	0.798	0.922	0.919
	HT2	0.925			
	HT3	0.846			
PI^h^	PI1	0.745	0.641	0.842	0.840
	PI2	0.764			
	PI3	0.886			
PPR^i^	PPR1	0.823	0.699	0.874	0.871
	PPR2	0.909			
	PPR3	0.771			
BI^j^	BI1	0.899	0.805	0.943	0.942
	BI2	0.933			
	BI3	0.906			
	BI4	0.849			

^a^AVE: average variance extracted, ^b^CR: composite reliability, ^c^PE: performance expectancy, ^d^EE: effort expectancy, ^e^SI: social influence, ^f^FC: facilitating conditions, ^g^HT: habit, ^h^PPR: perceived privacy risks, ^i^PI: Patients innovation, ^j^BI: behavioral intention

**Table 3:** Discriminant validity

Square root of AVE^a^	PE	EE	SI	FC	HT	PI	PPR	BI
PE^b^	0.944							
EE^c^	0.798	0.917						
SI^d^	0.807	0.875	0.847					
FC^e^	0.812	0.912	0.954	0.834				
HT^f^	0.793	0.863	0.901	0.956	0.893			
PI^g^	0.449	0.501	0.512	0.555	0.613	0.801		
PPR^h^	-0.106	-0.140	-0.126	-0.140	-0.114	-0.118	0.836	
BI^i^	0.621	0.686	0.674	0.709	0.756	0.638	-0.227	0.897

^a^AVE: average variance extracted, ^b^PE: performance expectancy, ^c^EE: effort expectancy, ^d^SI: social influence, ^e^FC: facilitating conditions, ^f^HT: habit, ^g^PPR: perceived privacy risks, ^h^PI: patients innovation, ^i^BI: behavioral intention

### The structural model

Table 4 shows the fitting index values of the structural model. GFI is less than the recommended value, but it is still within the acceptable range, and other fitting index values all meet the optimal condition, indicating that the collected data fits the structural model well. It can be seen from Table 5 that 4 hypotheses are rejected among the 11 hypotheses in this study, and the standardized regression coefficients of all other variables are significant.

**Table 4:** Fit indexes

Fit indexes	χ^2^/df	GFI^a^	NFI^b^	CFI^c^	RMSEA^d^	IFI^e^
Measurement model	2.631	0.874	0.930	0.956	0.064	0.956
Recommended value	< 3	> 0.9	> 0.9	> 0.9	< 0.08	> 0.9

^a^GFI: the goodness of fit index,^b^NFI: normed fit index, ^c^CFI: comparative fit index, ^d^RMSEA: the root mean-squared error of approximation, ^e^IFI: incremental fit index

**Table 5:** Standardized regression weights between the model variables

Path	β (95% CI)	*t*-value	*P*-value	Accept or reject the hypothesis
H1: PE^a^ → BI^h^	0.096 (-0.079, 0.275)	1.191	0.233	Reject
H2: EE^b^ → BI	0.239 (-0.022, 0.478)	1.980	0.048	Accept
H3: SI^c^ → BI	-0.011 (-1.146,1.715)	-0.032	0.975	Reject
H4:SI→PE	0.837 (0.772, 0.890)	18.624	< 0.001	Accept
H5: FC^d^ →BI	-0.251 (-2.881, 1.236)	-0.572	0.567	Reject
H6:FC→EE	0.913 (0.877, 0.943)	25.014	< 0.001	Accept
H7: HT^e^→ BI	0.593 (0.072, 1.944)	3.142	0.002	Accept
H8: PPR^f^ → BI	-0.138 (-0.225, -0.047)	-3.465	< 0.001	Accept
H9: PI^g^→BI	0.269 (0.002, 0.443)	4.480	< 0.001	Accept
H10: PI→EE	0.028 (-0.035, 0.099)	1.010	0.312	Reject
H11: PI→PPR	-0.113 (-0.235, 0.001)	-1.964	0.050	Accept

^a^PE: performance expectancy, ^b^EE: effort expectancy, ^c^SI: social influence, ^d^FC: facilitating conditions, ^e^HT: habit, ^f^PPR: perceived privacy risks, ^g^PI: patients innovation, ^h^BI: behavioral intention

**Fig. 2:**
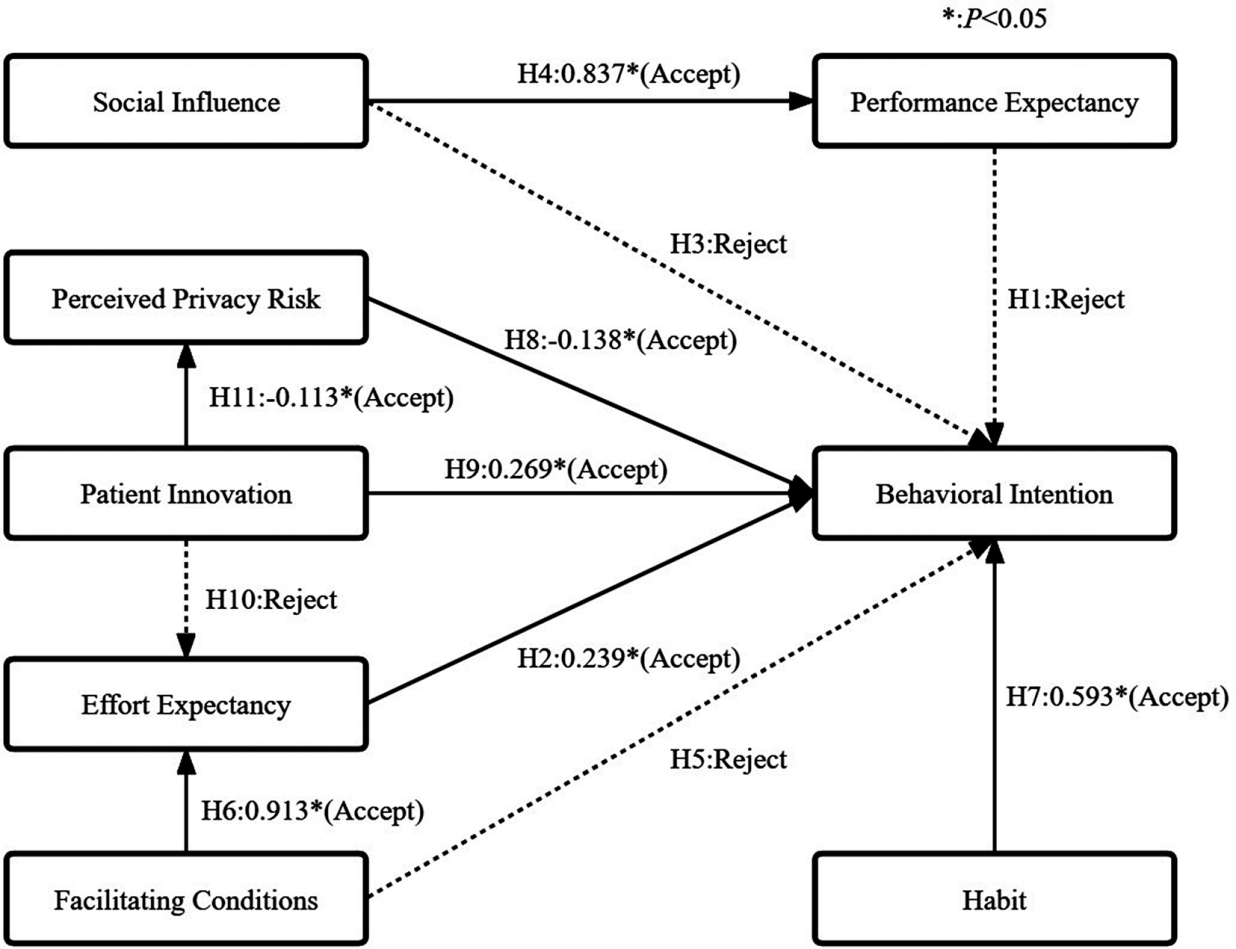
The results of model fitting

The result model is shown in Fig. 2. HT is the main positive factor influencing the BI of using HERS, followed by EE, PI, and PPR have a significantly negative effect on BI. PI has an indirect effect on BI (β = 0.016; 95%CI: 0.001, 0.051; *P* < 0.001), and PPR plays a mediating role in this indirect effect. FC have an indirect effect on BI (β = 0.218; 95%CI: 0.020, 0.440; *P* < 0.001), and EE plays a mediating role in this indirect effect. The above variables explained 56.7% of the variation in BI.

## Discussion

### Guide patients to use HERS and help them to develop habits

This study finds that patients with the habit of using a new system had a higher BI, which was consistent with the results of Baudier, and Zanetta et al. [29, 30] Nowadays, more and more hospitals begin to implement intelligent medical systems to improve medical quality and service. From the first contact with such new systems, patients will gradually get familiar with the use of them. With the passage of time (namely experience), patients can form different degrees of habits based on their interaction and familiarity with the system [14]. Ajzen et al. point out that feedback from experience can influence beliefs [31], so patients who are accustomed to using similar information systems are more likely to use the HERS. “Habit” is defined as a specific form of automaticity in which responses are directly cued by the contexts [32]. Therefore, patients would ignore the existence of the new system due to the habit, and they will habitually ask doctors for help making manual appointments and check-ins (manual registration is the old form of hospital), which leading to low utilization rate of HERS and high complaint rate of patients. Habits are hard to change, they take repetition to form [33], so it is important that managements arrange some volunteers to guide patients to use the new system. During intervention, it is also important to remind the patients to bring up the habit [34].

### Arrange staffs to solve the difficulties about using

FC refer to the resource condition for patients to use the HERS. In this study, FC do not directly affect the BI because HERS is free for using, and there will be staff helping solve the problems. Adequate convenience increases the ease of system using, which is consistent with previous studies [16, 20]. Patients will more like to use the system if they find HERS is easy to use. but PPR would discourage patients from using. To sum up, the hospital manageements should continue arranging staffs to solve the difficulties in using HERS, and they can reassure patients by introducing them with the system’s privacy capabilities. What’s more, it is necessary to continuously upgrade the system to make its operation clearer, simpler and more secure.

### Invite patients with high innovation to recommend HERS to others

The influence of innovation on BI has been confirmed by relevant studies [35–37], Thakur also pointed out that improving innovation will reduce the PPR of using a new system, which is consistent with our findings. In this study, innovation is a patient’s personal trait, which can be expressed as four levels of meaning, namely (1) hope: stay committed to goals and redirect path to the goals to achieve success when encountering difficulties, (2) self-efficacy: have confidence in taking on challenging and put in the effort necessary to succeed, (3) resilience: pick up and maintain or increase efforts to succeed when beset by adversity, and (4) optimism: has a positive feeling about future success [38, 39]. Patients with higher level of innovation have higher initiative than others, and lower frustration after failure. Therefore, such patients are more willing to use or continue to use the system. However, innovation is a personal characteristic, it is difficult to intervene in it. According to diffusion of innovations theory, the belief in the effectiveness may be more important than the actual results [40]. So it may be an effective way that managements invites patients with high innovation and doctors to recommend the new system, and patients with low innovation will value their opinions.

### Implement HERS with the way of parallel switching

PE and SI do not affect the BI, but the role of these two factors could not be ignored in related studies [19, 41, 42], we think the reasons could be as follows: (1) Although HERS can improve the examination efficiency, the improvement is mainly reflected by the data in HIS (comparing the average waiting time before and after using the HERS), and patient’s perception is not obvious; (2) Patients don’t come to the hospital very often. Although HERS has been implemented for six months, some patients are still new to the system; (3) Doctors and patients are still not proficient in using HERS, so they think learning to use HERS will add their troubles, and there are few cases of doctors recommending to patients and patients recommending to patients. Therefore, relevant training about HERS should be carried out among doctors before implementing the new system in hospitals. At the same time, strengthening testing and parallel switching should be adopted to pass the transition period to the new system [43, 44]. Parallel switching refers to the way of implementing the new system while retaining the old one, and patients will personally realize the advantages of HERS in parallel switching period and the system would be more acceptable.

### Limitations

First, the samples in this study were only from one 3 A hospital, so the representativeness is limited. Future multi-center studies can be carried out. Second, the data of this study came from questionnaires, and there may be bias in questionnaire survey. Third, potential moderators (age, gender et al.) which could affect patients’ behavior intention were not taken into consideration.

## Conclusions

This study aims to find out the factors that influencing patients’ BI of using HERS and provide valuable insights for hospital managements to drive the effective implementation of HERS. Through statistic analysis, HT, EE and PI were turned out to be the positive factors and PPR was turned out to be the negative factor affecting BI. So it is important to find the HERS implementation plan of promotion in these points. When HERS is implemented in hospitals, managements should arrange volunteers to guide patients to bring up the habit and solve the using difficulties, HERS needs to be continuously optimized to reduce the difficulty of use, and managements could invite patients with high innovation to recommend HERS to others, what’s more, it is a valid way to retain the old form of appointment to pass the transition period to the new system. HERS utilization and patients’ medical satisfaction will be enhanced through the guidance of hospital management means.

### Electronic supplementary material

Below is the link to the electronic supplementary material.

Supplementary Material 1

## Data Availability

The datasets used and/or analysed during the current study are available from the corresponding author on reasonable request.

## List of abbreviations

HIS: Hospital Information System
HERS: Hospital Examination Reservation System
UTAUT2: the Unified Theory of Acceptance and Use of Technology 2
PE: Performance Expectancy
EE: Effort Expectancy
SI: Social Influence
FC: Facilitating Conditions
HT: Habit
BI: Behavioral Intention
PI: Patients Innovation
PPR: Perceived Privacy Risks
